# Size and Proportions of Slow-Twitch and Fast-Twitch Muscle Fibers in Human Costal Diaphragm

**DOI:** 10.1155/2016/5946520

**Published:** 2016-11-07

**Authors:** Marija Meznaric, Erika Cvetko

**Affiliations:** Institute of Anatomy, Faculty of Medicine, University of Ljubljana, Korytkova 2, Sl-1000 Ljubljana, Slovenia

## Abstract

Smaller diaphragmatic motor unit potentials (MUPs) compared to MUPs of limb muscles lead to the hypothesis that diaphragmatic muscle fibers, being the generators of MUPs, might be also smaller. We compared autopsy samples of costal diaphragm and vastus lateralis of healthy men with respect to fibers' size and expression of slow myosin heavy chain isoform (MyHC-1) and fast 2A isoform (MyHC-2A). Diaphragmatic fibers were smaller than fibers in vastus lateralis with regard to the mean minimal fiber diameter of slow-twitch (46.8 versus 72.2 *μ*m, *p* < 0.001), fast-twitch (45.1 versus 62.4 *μ*m, *p* < 0.001), and hybrid fibers (47.3 versus 65.0 *μ*m, *p* < 0.01) as well as to the mean fiber cross-sectional areas of slow-twitch (2376.0 versus 5455.9 *μ*m^2^, *p* < 0.001), fast-twitch (2258.7 versus 4189.7 *μ*m^2^, *p* < 0.001), and hybrid fibers (2404.4 versus 4776.3 *μ*m^2^, *p* < 0.01). The numerical proportion of slow-twitch fibers was higher (50.2 versus 36.3%, *p* < 0.01) in costal diaphragm and the numerical proportion of fast-twitch fibers (47.2 versus 58.7%, *p* < 0.01) was lower. The numerical proportion of hybrid fibers did not differ. Muscle fibers of costal diaphragm have specific characteristics which support increased resistance of diaphragm to fatigue.

## 1. Introduction

Diaphragm, a principal inspiratory muscle of humans, is a highly specialized skeletal muscle unique in its ability to contract continuously and rhythmically. Quantitative motor unit potential (MUP) analysis in healthy volunteers has established that amplitude, area, and size index of MUPs are much smaller in costal diaphragm than in limb muscles [1]. Muscle fiber size is one of the variables which contribute to the amplitude of MUPs [2], suggesting that muscle fibers of costal diaphragm, being the generators of MUPs, might also be smaller than those of limb muscles. Studies about muscle fiber diameters in human costal diaphragm are sparse and contradictory quoting similar [3], smaller [4, 5], or bigger [6] size of diaphragmatic fibers with respect to limb muscle fibers. The aim of the present study was to establish normative morphometric data of muscle fibers in human costal diaphragm with respect to the size of slow-twitch and fast-twitch muscle fibers and their numerical proportions.

## 2. Materials and Methods

The muscle sampling was approved by the National Medical Ethics Committee of the Republic of Slovenia (permission number 36/04/08). Postmortem costal diaphragm was sampled bilaterally, in the midclavicular line, near the attachment of diaphragmatic fibers to the costal arch. Left vastus lateralis was sampled 15 cm above the patella. Muscle samples were collected 7–17 hours after death (mean ± SD; 12.6 ± 3 hours), from 16 healthy males aged 23–59 years (mean ± SD; 43.3 ± 3 years) who died accidently.

Muscle fibers were classified according to the expression of myosin heavy chain isoforms (MyHC) by indirect immunoperoxidase method as described previously [7]; briefly,* slow* fibers were demonstrated by BA-D5 antibody immunoreactive with *β*/slow MyHC-I in rats [8] and humans [9];* fast *fibers were demonstrated by A4.74 antibody (former Alexis Biochemicals, now Enzo Life Sciences) immunoreactive with MyHC-IIA and MyHC-IIX in humans [7]. If fibers were immunoreactive with both antibodies, they were labeled as* hybrid* fibers. BA-D5 antibody was produced from mouse hybridoma BA-D5 cell line provided by Deutsche Sammlung von Mikroorganismen und Zellkulturen (DSMZ, Braunschweig, Germany). BA-D5 and A4.74 antibodies were diluted 1 : 100 in PBS/BSA and detected with rabbit anti-mouse immunoglobulins, peroxidase conjugated (Dako, Denmark).

Images of 10 *μ*m serial frozen muscle sections stained by the BA-D5 or A4.74 antibodies were captured by Nikon Eclipse 8000 microscope equipped with Nikon digitalized camera DXM 1200F and computer software for image acquisition (Lucia GF software, version 4.82, Laboratory imaging, Prague, Czech Republic). Outlining contour of individual slow, fast, and intermediate muscle fibers was performed by commercial image analysis program Ellipse (ViDiTo, Kosice, Slovakia). On average 400 muscle fibers were analyzed in costal diaphragms on each side and 230 fibers in each sample of vastus lateralis.

Average* minimal* muscle fiber diameters, average fiber cross-sectional areas, and numerical proportions of muscle fibers were calculated for each individual sample (right and left side of the costal diaphragm and vastus lateralis) by software for muscle fiber type classification and analysis [10]. Statistical analysis was performed with the statistical package SYSTAT version 5.0 for Windows. Paired samples *t*-test was used to test the difference between the right and left side of the costal diaphragm and independent samples *t*-test to test the differences between the costal diaphragm and vastus lateralis muscle.

## 3. Results

### 3.1. Symmetrical Sides of Costal Diaphragm

The right and left side of costal diaphragm did not differ significantly (*p* > 0.05) (Table 1) in the mean minimal fiber diameters, the mean fiber cross-sectional areas, and the numerical proportions of slow-twitch, fast-twitch, and hybrid fibers.

### 3.2. Costal Diaphragm Compared to Vastus Lateralis

**Figure 1:**
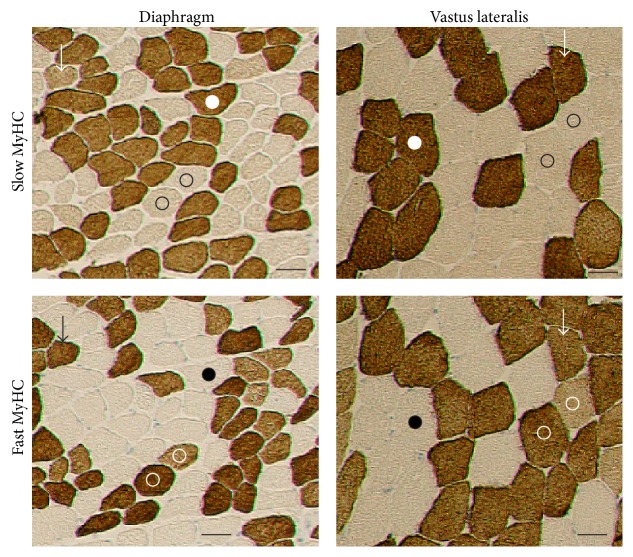
Phenotyping of muscle fibers by the expression of slow and fast MyHCs in costal diaphragm and vastus lateralis muscle. Fibers labeled by dots are slow-twitch fibers, fibers labeled by open circles are fast-twitch fibers, and hybrid fibers are labeled by arrows. Muscle fibers not stained by antibodies to slow MyHC are stained heavily or intermediately by antibodies to fast MyHC. Bar = 50 *μ*m.

Muscle fibers of costal diaphragm were smaller than muscle fibers of vastus lateralis (Figure 1).

Mean muscle fiber diameter and mean muscle fiber cross-sectional area of all fiber types in costal diaphragm were smaller than in vastus lateralis, *p* < 0.001 for slow-twitch and fast-twitch fibers; *p* < 0.01 for hybrid fibers expressing MyHC-1 and MyHC-2A (Table 2). The mean fiber diameters and the mean fiber cross-sectional areas were similar among different fiber types of costal diaphragm. The costal diaphragm contained on average about the same numerical proportion of slow-twitch and fast-twitch fibers which is different (*p* < 0.01) from vastus lateralis (Table 2). The mean numerical proportion of fast-twitch fibers in costal diaphragm was significantly lower (*p* < 0.01) than in vastus lateralis and the mean numerical proportion of hybrid fibers did not differ significantly (*p* > 0.05).

**Figure 2:**
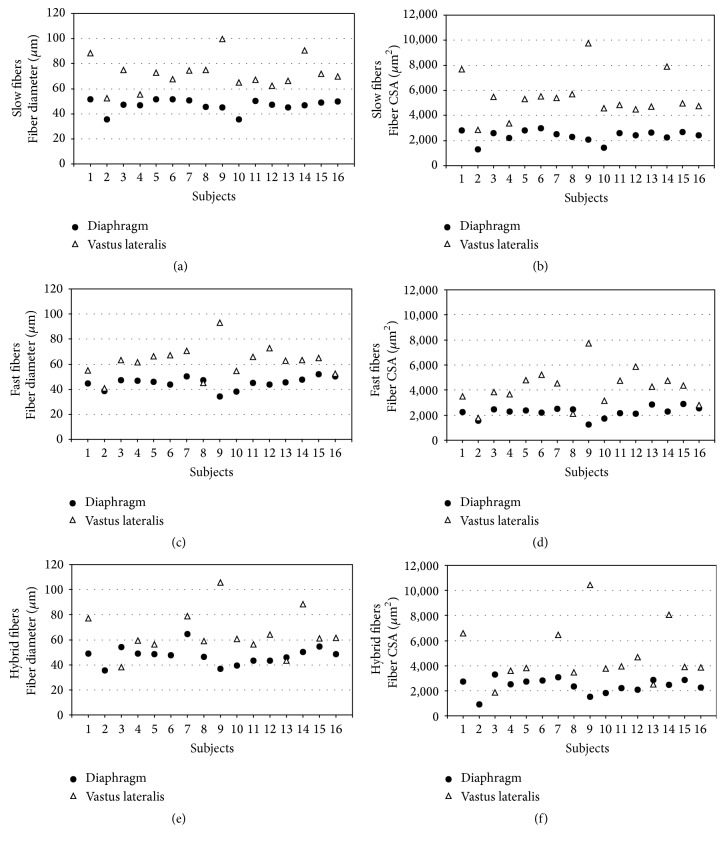
Variability between subjects with regard to fiber diameters and fiber cross-sectional areas. CSA = cross-sectional area. Slow-twitch fibers (a, b) of costal diaphragm were smaller with regard to diameters and fiber cross-sectional areas than those of vastus lateralis muscle in all subjects. Fast-twitch fibers of costal diaphragm (c, d) were in 13/16 subjects smaller and in 3/16 subjects of similar size compared to those of vastus lateralis. Hybrid fibers (e, f) of costal diaphragm were in 12/16 subjects smaller, in 1/16 of similar size, and in 1/16 bigger than those of vastus lateralis. In 2/16 comparison was not possible, since hybrid fibers were absent in vastus lateralis muscle.

**Figure 3:**
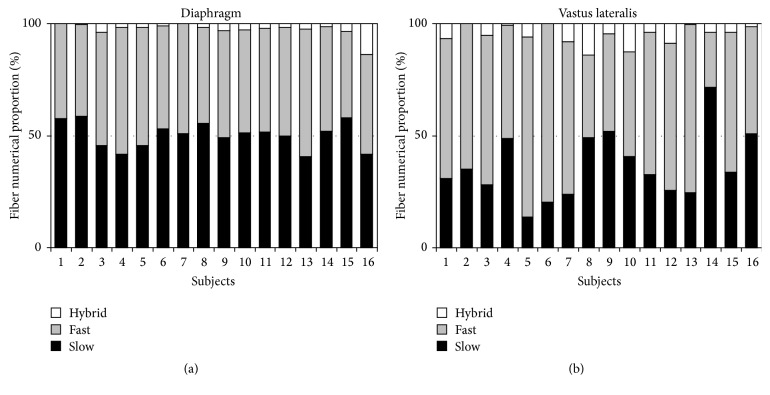
Variability between subjects with regard to the numerical proportions of fiber types. Numerical proportion of slow-twitch fibers of costal diaphragm (a) was in all subjects around 50%, while in vastus lateralis muscle (b) this was the case only in 4/16 subjects and in the majority (11/16) slow-twitch fibers constitute about one-third of muscle fibers of vastus lateralis. In 1/16 subjects the proportion of slow-twitch fibers was around 70%. The proportion of hybrid fibers was small but varied considerably in both muscles; in costal diaphragm minimum was 0.1% and maximum 13.4% and in vastus lateralis minimum was 0.0% and maximum 14%.

The variability between subjects with regard to the fiber size is illustrated in Figure 2 and with regard to the numerical proportions of fiber types in Figure 3.

## 4. Discussion

The present investigation is based on the visualization of muscle fibers types by the expression of MyHC isoforms.

In human muscles 2A fibers are heavily stained with antibodies directed against MyHC-2A (A4.74 antibody) and 2X fibers stain intermediately [7, 11] which is similar to immunoreactivity of this antibody in dogs [12] but different from rats [7] in which this antibody stains only 2A fibers. On muscle sections fibers not immunoreactive with anti-slow MyHC-1 antibodies were all stained either heavily or intermediately with A4.74 antibodies which is in agreement that this antibody can be considered as an anti-fast myosin in humans (Figure 1) [7, 11].

We have demonstrated that slow-twitch fibers (expressing MyHC-1), fast-twitch fibers (expressing MyHC-2A), and hybrid muscle fibers (expressing MyHC-1 and MyHC-2A) of costal diaphragm were smaller than the corresponding muscle fibers of vastus lateralis muscle. With the use of anti-fast MyHC and anti-slow MyHC antibodies, we were not able to distinguish between hybrid fibers expressing both types of fast myosin 2A and 2X from pure 2A fibers; neither could we directly demonstrate eventual pure 2X fibers. As the small size of diaphragmatic fibers is important for physiological characteristics of diaphragmatic fibers (see below), probably also other subtypes of fast-twitch diaphragmatic fibers are smaller in size than those of vastus lateralis muscle, but this has to be demonstrated in further studies.

For the sake of comparison with other studies, we calculated two parameters for the estimation of fiber size, mean minimal fiber diameter, and mean fiber cross-sectional area. Theoretically, the minimal muscle fiber diameter should be a more reliable estimate of muscle fiber size than cross-sectional area, since it is independent of the cutting angle during sample processing [13]. Nevertheless, several studies describing diaphragmatic fibers in different physiological conditions in animals [14, 15] or pathological states in humans [6, 16, 17] are actually operating with mean fiber cross-sectional area. If the muscle samples are large enough to allow appropriate orientation before cutting, as usually is fulfilled in autopsy studies and in animal studies, the selection of the parameter does not matter; but in analyzing small biopsy samples, particularly needle biopsies, the selection of the parameter could be of importance. Appropriate orientation of the small biopsy sample is a difficult task even for the experienced technician. Oblique sectioning and consequently overestimation of muscle fiber size by mean fiber cross-sectional area can practically not be avoided. Oblique sectioning might be the reason for the high mean muscle cross-sectional area of the diaphragmatic fibers, exceeding the mean fiber cross-sectional area of the limb muscle in controls, as reported by Levine et al. [6]. His results are in contradiction with the earlier studies [4, 5] and the present study, which all demonstrated smaller muscle fibers in human costal diaphragm compared to muscle fibers of limb muscle. Welvaart et al. [17] did not compare diaphragmatic fibers and muscle fibers of limb muscles but reported on absolutely larger mean muscle cross-sectional area of diaphragmatic fibers than the present study. The discrepancy between the studies may be partly explained by the different type of material analyzed in this study, autopsy material (which is prone to shrinkage due to dehydration) versus biopsy material in the study of Welvaart et al. [17]. Moreover the discrepancy might be partly due to the small number of subjects enrolled in the biopsy study which may be critical because of the interindividual variability. Since muscle fiber size is one of the variables which contributes to the amplitude of MUPs [2], circumstantial evidence for the smaller size of the diaphragmatic fibers comes also from MUP analysis of costal diaphragm which demonstrated smaller amplitude of diaphragmatic MUPs compared to those of limb muscles [1]. The relation between MUPs amplitude and muscle fiber diameter is valid for slow fibers only as the first recruited MUPs recorded by EMG arise from slow conducting motor units while later recruited, fast motor units, are not possible of being analyzed by EMG [18]. The small size of fast fibers of costal diaphragm does not have a direct support from MUP analysis. Nevertheless Mizuno [4] and this study have demonstrated that slow and fast diaphragmatic fibers are of similar size.

Interindividual variability of the size and numerical proportions of slow-twitch and fast-twitch muscle fibers was greater in vastus lateralis than in costal diaphragm. This could be explained by the well-known influence of the diverse life style practices regarding physical activity on muscle fiber size and composition of limb muscles [19]. In three individuals in whom the size of fast-twitch fibers was similar in vastus lateralis and costal diaphragm (Figure 2), this was not due to larger diaphragmatic fibers but to lower mean fiber diameter of fast-twitch fibers of vastus lateralis which is compatible with low level of physical activity in these subjects. Continuous and rhythmic activity of diaphragm on the other hand seems to suggest that no real disuse can occur in diaphragm in physiologic conditions [5]. This reflects in a more homogeneous diaphragmatic fiber population. Hybrid fibers expressing MyHC-1 and MyHC-2A were present in low percentage and varied considerably in both muscles. They are present in normal muscles and become more numerous during muscle fiber type transformation in response to exercise [20].

Increased resistance of diaphragm to fatigue [21] is a well-known characteristic of diaphragm. Two findings of this study are relevant in this respect: small size of diaphragmatic muscle fibers and high proportion of slow fibers which are known to be resistant to fatigue [5]. The small size of the diaphragmatic fibers reduces oxygen diffusion distance and (assuming the same capillarity as in limb muscle) makes oxygen supply to diaphragm more efficient [4, 22]. Similar [3] or even bigger sized [6, 17] diaphragmatic fibers would not be able to mediate such an effect under the same capillarity. The study of Sauleda et al. [3] was actually performed in patients showing mild airflow limitation and some air trapping consequently; they could not be considered as normative values.

We observed an overall lower numerical proportion of fast fibers in diaphragm compared to vastus lateralis but we did not subclassify fast fibers. As subtypes of fast fibers have different resistance to fatigue [5] it is quite possible that numerical proportions of subtypes of fast fibers, that is, fibers expressing MyHC-2A and MyHC-2X and hybrid fibers expressing both MyHC-2A and MyHC-2X, would be different in costal diaphragm. Theoretically the lowest proportion of the most fatigable (2X) fast-twitch fibers and the highest proportion of the most resistant fast-twitch fibers to fatigue (2A) would be expected (with an overall reduction of numerical proportion of fast-twitch fibers).

Similar mean fiber diameters, mean fiber cross-sectional areas, and fiber type proportions proved for the right and left side of the diaphragm are expected and related to symmetric contractions of diaphragm in normal physiological conditions.

## 5. Conclusions

Slow-twitch fibers (expressing MyHC-1), fast-twitch fibers (expressing MyHC-2A), and hybrid fibers (expressing MyHC-1 and MyHC-2A) of costal diaphragm had a lesser mean fiber diameters and a lesser mean fiber cross-sectional areas than muscle fiber types of vastus lateralis; however, subtypes of fast fibers were not investigated in this study.

The mean fiber diameters and the mean fiber cross-sectional areas were similar among the slow, fast, and intermediate fiber types (expressing MyHC-1 and MyHC-2A) of costal diaphragm.

The costal diaphragm contained about the same numerical proportion of slow-twitch and fast-twitch fibers which is different from vastus lateralis.

The mean numerical proportion of slow-twitch fibers was higher in costal diaphragm than in vastus lateralis muscle.

Smaller slow diaphragmatic muscle fibers might contribute to the smaller amplitude of diaphragmatic MUPs.

The small size of diaphragmatic muscle fibers (consequently short diffusion distance for oxygen) and high proportion of slow (fatigue resistant) fibers support increased resistance of diaphragm to fatigue.

## Figures and Tables

**Table 1:** Mean minimal fiber diameter, mean fiber cross-sectional area, and numerical proportion of slow-twitch, fast-twitch, and hybrid fibers of the right and left side of costal diaphragm.

Parameter	R slow	L slow	R fast	L fast	R hybrid	L hybrid
Diameter [*µ*m]	48.1 ± 2.3	45.8 ± 1.4	46.3 ± 2.1	45.4 ± 1.2	50.8 ± 2.6	46.0 ± 2.7
CSA [*µ*m^2^]	2502.6 ± 225.8	2248.7 ± 146.9	2321.3 ± 182.7	2225.5 ± 130.6	2891.5 ± 227.6^*∗*^	2314.9 ± 193.8^*∗*^
Numerical proportion [%]	51.3 ± 2.9	49.3 ± 1.3	47.2 ± 2.8	49.2 ± 1.1	1.5 ± 0.4^*∗∗*^	1.5 ± 0.4^*∗∗*^

Values are means ± SE. CSA = cross-sectional area; R = right side of costal diaphragm; L = left side of costal diaphragm; slow = slow-twitch fibers; fast = fast-twitch fibers; hybrid = hybrid fibers; *N* = 9 (*p* > 0.05 in all, ^*∗*^ *p* minimal = 0.180, and ^*∗∗*^ *p* maximal = 0.974).

**Table 2:** Comparison of costal diaphragm and vastus lateralis muscle with regard to mean fiber diameters, mean fiber cross-sectional areas, and mean numerical proportions of fiber types.

Parameter	DIA slow	VL slow	DIA fast	VL fast	DIA hybrid	VL hybrid
Diameter [*µ*m] CV [%]	46.8 ± 1.2^*∗*^ 10.5	72.2 ± 3.1^*∗*^ 17.2	45.1 ± 1.2^*∗*^ 10.4	62.4 ± 3.0^*∗*^ 19.3	47.3 ± 1.8^*∗∗*^ 15.0	65.0 ± 4.7^*∗∗*^ 27.0
CSA [*µ*m^2^] CV [%]	2376.0 ± 115.9^*∗*^ 19.5	5455.2 ± 428.0^*∗*^ 31.4	2258.7 ± 110.6^*∗*^ 19.4	4189.7 ± 362.5^*∗*^ 34.6	2404.44 ± 151.4^*∗∗*^ 25.2	4776.32 ± 618.8^*∗∗*^ 48.5
Numerical proportion [%] CV [%]	50.2 ± 1.5^*∗*^ 11.6	36.3 ± 3.7^*∗*^ 41.0	47.2 ± 1.3^*∗*^ 10.9	58.7 ± 3.9^*∗*^ 26.7	2.6 ± 0.8 122.5	5.0 ± 1.1 85.0

Values are means ± SE. CV = coefficient of variation; CSA = cross-sectional area; DIA = costal diaphragm; VL = vastus lateralis; slow = slow-twitch fibers; fast = fast-twitch fibers; hybrid = hybrid fibers; *N* = 16 (^*∗*^ *p* < 0.001; ^*∗∗*^ *p* < 0.01).
